# Predatory Bacteria: A Potential Ally against Multidrug-Resistant Gram-Negative Pathogens

**DOI:** 10.1371/journal.pone.0063397

**Published:** 2013-05-01

**Authors:** Daniel E. Kadouri, Kevin To, Robert M. Q. Shanks, Yohei Doi

**Affiliations:** 1 Department of Oral Biology, University of Medicine and Dentistry of New Jersey, Newark, New Jersey, United States of America; 2 Department of Ophthalmology, Campbell Laboratory of Ophthalmic Microbiology, University of Pittsburgh, Pittsburgh, Pennsylvania, United States of America; 3 Division of Infectious Diseases, University of Pittsburgh School of Medicine, Pittsburgh, Pennsylvania, United States of America; Institut National de la Recherche Agronomique, France

## Abstract

Multidrug-resistant (MDR) Gram-negative bacteria have emerged as a serious threat to human and animal health. *Bdellovibrio* spp. and *Micavibrio* spp. are Gram-negative bacteria that prey on other Gram-negative bacteria. In this study, the ability of *Bdellovibrio bacteriovorus* and *Micavibrio aeruginosavorus* to prey on MDR Gram-negative clinical strains was examined. Although the potential use of predatory bacteria to attack MDR pathogens has been suggested, the data supporting these claims is lacking. By conducting predation experiments we have established that predatory bacteria have the capacity to attack clinical strains of a variety of ß-lactamase-producing, MDR Gram-negative bacteria. Our observations indicate that predatory bacteria maintained their ability to prey on MDR bacteria regardless of their antimicrobial resistance, hence, might be used as therapeutic agents where other antimicrobial drugs fail.

## Introduction

Since antimicrobial drugs were first discovered they have saved countless lives. However, pathogenic multidrug-resistant (MDR) bacteria have emerged as a serious threat to human health. Of particular concern are MDR Gram-negative bacteria producing highly potent ß-lactamases such as the extended-spectrum ß-lactamase and KPC-type ß-lactamase [1]. It is estimated that in the United States alone nearly 2 million patients develop hospital-acquired infection yearly [2], many of which are caused by these MDR pathogens. The magnitude of the problem has highlighted the need to develop new ways to control infection.

An alternative approach to combat antimicrobial-resistant bacterial infections is the use of predatory bacteria to eliminate MDR pathogens. *Bdellovibrio* spp. and *Micavibrio* spp. are Gram-negative bacteria which belong to the delta and alpha subgroup of proteobacteria respectively [3], [4]. The *Bdellovibrio* life cycle involves attack phase cell that seek, attach to, and invade a Gram-negative bacterial host, and a growth phase cell that develops within the host [5]–[7]. The *Micavibrio* life cycle also exhibits an attack phase cell that allows it to find its Gram-negative bacterial host and to attach to the prey's surface, followed by extracellular growth of the predator [8]–[10]. We have previously demonstrated that both *Bdellovibrio* and *Micavibrio* have the potential to prey on a wide range of human pathogens grown both planktonically and as a biofilm [11]–[13]. However, the majority of the studies utilized culture collection reference strains or clinical strains for which the antibiotic susceptibility data were lacking [11], [13]. Therefore, the ability of predator bacteria to attack contemporary clinical strains of MDR bacteria has remained unclear. To address this question, we examined the capacity of the two predatory bacteria to prey on MDR Gram-negative clinical strains producing clinically relevant ß-lactamases and representing various opportunistic nosocomial pathogens.

## Materials and Methods

A total of 14 MDR clinical strains isolated between 2005 and 2011 were tested, including *Acinetobacter baumannii* [2], *Escherichia coli* [5], *Klebsiella pneumoniae* [5], and *Pseudomonas* spp. [2]. They were selected to include species which are commonly encountered clinically, and to represent a variety of potent ß-lactamases, including extended-spectrum ß-lactamase (ESBL), KPC-type carbapenemase, AmpC-type ß-lactamase, and metallo-ß-lactamase. Antimicrobial susceptibility was tested using the disk diffusion method and interpreted according to the breakpoints endorsed by the Clinical and Laboratory Standards Institute (CLSI) (Table 1) [14]. The ß-lactamases produced were characterized previously [15], [16] or otherwise determined by PCR and sequencing [17]. Three predatory bacteria were used in this study: *Bdellovibrio bacteriovorus* 109J (ATCC 43826), *B. bacteriovorus* HD100 and *Micavibrio aeruginosavorus* strain ARL-13 [5], [10]. The predators were grown and maintained as described before [11]. Predator stock-lysates were made by co-culturing host cells with the predators in diluted nutrient broth (DNB) and allowing the co-culture to incubate at 30°C on a rotary shaker until the culture became clear. To culture the predators, co-cultures were prepared by adding 2 ml of washed host cells (∼1×10^9^ CFU/ml) to 2 ml of predatory bacteria stock-lysate in 20 ml of DNB. The co-cultures were incubated for 24 hrs until the predator reached a final concentration of ∼1×10^8^ PFU/ml. Thereafter, the lysates were filtered through a 0.45-µm Millex pore-size filter (Millipore, Billerica, MA) in order to remove remaining host cells (predator filtered lysate). As a control, filtered sterilized lysate was prepared by passing the lysates through three 0.22 µm pore-size filters [12], [13]. Predation experiments were conducted as described previously [11]. In brief, 5 ml of DNB co-cultures were made by adding to 0.5 ml of washed host cells to 0.5 ml of predator filtered lysate or predator-free control. The cultures were placed at 30°C on a rotary shaker for 48 hrs.

**Table 1:** Host pathogens used in the study and their antibiotic susceptibility.

Bacteria and strain	Source	ß-lactamase gene	Antibiotic susceptibility
***Acinetobacter baumannii***			
AB276	Sputum	OXA-23	Ceftazidime (R); Cefotaxime (R); Cefepime (S); Imipenem (I); Meropenem (R); Gentamicin (R); Amikacin (R); Ciprofloxacin (R); Tetracycline (R); Trimethoprim-sulfamethoxazole (R)
AB285	Donor bronchus	OXA-40	Ceftazidime (R); Cefotaxime (R); Cefepime (R); Imipenem (R); Meropenem (R); Gentamicin (R); Amikacin (R); Ciprofloxacin (R); Tetracycline (R); Trimethoprim-sulfamethoxazole (R)
***Escherichia coli***			
YD429	Urine	CTX-M-15	Ceftazidime (I); Cefotaxime (R); Cefepime (S); Imipenem (S);Meropenem (S); Gentamicin (R); Amikacin (S); Ciprofloxacin (R);Tetracycline (R); Trimethoprim-sulfamethoxazole (R)
YD438	Blood	SHV-7	Ceftazidime (S); Cefotaxime (S); Cefepime (S); Imipenem (S); Meropenem (S); Gentamicin (S); Amikacin (S); Ciprofloxacin (R); Tetracycline (R); Trimethoprim-sulfamethoxazole (S)
YD446	Urine	CTX-M-14	Ceftazidime (S); Cefotaxime (R); Cefepime (S); Imipenem (S); Meropenem (S); Gentamicin (S); Amikacin (S); Ciprofloxacin (R); Tetracycline (R); Trimethoprim-sulfamethoxazole (R)
YDC354	Urine	KPC-2	Ceftazidime (R); Cefotaxime (R); Cefepime (S); Imipenem (R); Meropenem (R); Gentamicin (S); Amikacin (S); Ciprofloxacin (R); Tetracycline (R); Trimethoprim-sulfamethoxazole (R)
AZ1285	Blood	CMY-33	Ceftazidime (R); Cefotaxime (R); Cefepime (R); Imipenem (S); Meropenem (S); Gentamicin (S); Amikacin (S); Ciprofloxacin (R); Tetracycline (R); Trimethoprim-sulfamethoxazole (S)
***Klebsiella pneumoniae***			
YD466	Wound	KPC-2	Ceftazidime (R); Cefotaxime (R); Cefepime (R); Imipenem (R); Meropenem (R); Gentamicin (S); Amikacin (R); Ciprofloxacin (R); Tetracycline (S); Trimethoprim-sulfamethoxazole (R)
AZ1032	Blood	SHV-7	Ceftazidime (R); Cefotaxime (R); Cefepime (S); Imipenem (S); Meropenem (S); Gentamicin (R); Amikacin (S); Ciprofloxacin (S); Tetracycline (S); Trimethoprim-sulfamethoxazole (R)
AZ1093	Blood	SHV-5	Ceftazidime (R); Cefotaxime (R); Cefepime (I); Imipenem (S); Meropenem (S); Gentamicin (R); Amikacin (R); Ciprofloxacin (S); Tetracycline (S); Trimethoprim-sulfamethoxazole (R)
AZ1136	Blood	CTX-M-2	Ceftazidime (R); Cefotaxime (R); Cefepime (R); Imipenem (S); Meropenem (I); Gentamicin (R); Amikacin (R); Ciprofloxacin (R); Tetracycline (S); Trimethoprim-sulfamethoxazole (R)
AZ1169	Blood	SHV-12	Ceftazidime (R); Cefotaxime (R); Cefepime (S); Imipenem (S); Meropenem (S); Gentamicin (S); Amikacin (S); Ciprofloxacin (R); Tetracycline (S); Trimethoprim-sulfamethoxazole (R)
***Pseudomonas aeruginosa***			
GB771	Sputum	PME-1	Ceftazidime (R); Cefotaxime (R)^1^; Cefepime (R); Imipenem (R); Meropenem (R); Gentamicin (R); Amikacin (S); Ciprofloxacin (R); Tetracycline (R)^1^; Trimethoprim-sulfamethoxazole (R)^1^
***Pseudomonas putida***			
YA241	Sputum	VIM-1	Ceftazidime (R); Cefotaxime (R)^1^; Cefepime (R); Imipenem (R); Meropenem (R); Gentamicin (R); Amikacin (S); Ciprofloxacin (R); Tetracycline (I)^1^; Trimethoprim-sulfamethoxazole (R)^1^

(R) Resistant; (I) intermediate; (S) susceptible.

1 Breakpoints are not defined for cefotaxime, tetracycline and trimethoprim-sulfamethoxazole by the CLSI; interpretation based on the breakpoints for *A. baumannii*.

## Results and Discussion

The ability of each predator to attack the host was measured by the reduction in host cell viability, determined by dilution plating and CFU enumeration, and compared to the initial host concentration and predator-free control. Cell viability was measured following 24 and 48 hrs of incubation. Each co-culture was performed in triplicate. The ability of the predators to attack each of the MDR pathogens is shown in Table 2. *B. bacteriovorus* HD100 was able to prey on all examined host bacteria with a greater than 2, 3 and 4 log_10_ reduction measured for 93%, 78% and 35% of the attacked strains, respectively. *B. bacteriovorus* 109J was able to prey on 13 of the 14 host bacteria (93%) with a greater than 2, 3 and 4 log_10_ reduction measured for 85%, 64% and 28% of the predation positive strains, respectively. Five out of the 7 (71%) examined host bacteria were reduced by *M. aeruginosavorus* ARL-13, with 80% and 40% of the predator-susceptible strains showing a 2 and 3 log_10_ reduction, respectively. In this study *Micavibrio* was examined only on *P. aeruginosa* and *K. pneumoniae* as previous study suggested that *M. aeruginosavorus* ARL-13 is most capable of preying on these pathogens [11], [13]. The predators maintained their ability to prey on the host cells despite the MDR status. Furthermore, no clear patterns emerged when comparing the antibiotic susceptibility of the host cells to predation. The different host specificity observed for each predator, as well as the differential capacity of each predator strain to prey on certain stains within the same species, is well documented for both *Bdellovibrio* and *Micavibrio* [6], [9], [11], [13], [18]–[20]. However, as the mechanisms that govern host specificity are not fully understood, it is difficult to speculate on the reason way some host strains are consumed by the predators whereas others are not.

**Table 2:** Change in host viability following predation.

Bacteria and strain	Time_0_ (CFU/ml)	Control (Log_10_ change)	*B. bacteriovorus* 109J (Log_10_ change)	*B. bacteriovorus* HD100 (Log_10_ change)	*M. aeruginosavorus *ARL-13 (Log_10_ change)
***Acinetobater baumannii***					
AB276	3.38×10^8^	+0.47±0.21	−3.92±0.27	−3.79±0.07	na
AB285	2.50×10^8^	+0.12±0.28	−3.56±0.06	−2.75±0.11	na
***Escherichia coli***					
YD429	3.13×10^8^	+0.03±0.02	−1.7±0.20	−3.68±0.11	na
YD438	1.38×10^8^	+0.09±0.51	−3.55±0.20	−3.89±0.84	na
YD446	4.50×10^8^	+0.07±0.03	−2.96±0.18	−3.2±0.3*	na
YDC354	4.25×10^8^	+0.01±0.11	−0.1±0.12*^Ψ^	−3.72±0.07	na
AZ1285	6.00×10^8^	+0.80±1.13	−3.61±0.07	−3.8±0.84	na
***Klebsiella pneumoniae***					
YD466	3.63×10^8^	+0.24±0.18	−3.99±0.36	−3.73±0.20	−2.91±0.19
AZ1032	4.38×10^8^	+0.07±0.05	−2.75±0.10	−4.04±0.56	−0.05±0.07^Ψ^
AZ1093	4.30×10^8^	+0.28±0.23	−2.42±0.07	−4.09±0.39	−0.70±0.11^Ψ^
AZ1136	4.63×10^8^	+0.08±0.03	−3.54±0.36	−2.83±0.09	−3.01±0.19
AZ1169	4.61×10^8^	−0.43±0.20	−4.51±0.55	−1.79±0.15	−2.85±0.05*
***Pseudomonas aeruginosa***					
GB771	2.53×10^8^	+0.71±0.26	−3.96±0.32*	−3.07±0.68	−2.64±0.29
***Pseudomonas putida***					
YA241	6.25×10^6^	+0.94±0.22	−2.4±0.14	−3.90±0.35	−1.41±0.35

Co-cultures were prepared by adding host cells to harvested predator cells (∼1×10^7^ PFU) or predator free control. Values represent the maximum log_10_ change measured following 24 or 48 (*) hrs of incubation (compared to t_0_). Each experiments was conducted in triplicates with value representing the mean and standard error.

**n.a**- not applicable.

**Ψ** =  experiment was conducted twice yielding similar result.

**Time 0**- initial host concentration (CFU/ml).

**+** =  Increase in host numbers.

**−** =  Decrease in host numbers.

## Conclusions

With the increased occurrence of MDR pathogens, many of which can no longer be treated adequately by conventional antimicrobial agents, becoming a major clinical concern, the concept of using predatory bacteria as live antimicrobials is gaining momentum [21]–[23]. Although the putative ability of predatory bacteria to attack MDR pathogens was hypothesized, it was never clearly demonstrated. Our data confirms that predatory bacteria maintained their ability to prey on MDR bacteria regardless of their antimicrobial resistance. This study further highlight the potential application of predatory bacteria as a biological control agent with the capability to prey on MDR Gram-negative pathogens which are currently found in clinical settings.
